# The Use of Flaps and Grafts in the Treatment of Urethral Stricture Disease

**DOI:** 10.1155/2015/979868

**Published:** 2015-11-19

**Authors:** Eric S. Wisenbaugh, Joel Gelman

**Affiliations:** University of California, Irvine, 333 City Boulevard West, Suite No. 1240, Orange, CA 92868, USA

## Abstract

The use of various grafts and flaps plays a critical role in the successful surgical management of urethral stricture disease. A thorough comprehension of relevant anatomy and principles of tissue transfer techniques are essential to understanding the appropriate use of grafts or flaps to optimize outcomes. We briefly review these principles and discuss which technique may be best suited for a given anterior urethral stricture, depending on the location and length of the stricture, the presence or absence of an intact corpus spongiosum, and the availability of adequate and healthy penile skin.

## 1. Introduction

### 1.1. Principles of Tissue Transfer

The two broad categories of tissue transfer are flaps and grafts. A flap refers to tissue that is transferred with its native blood supply intact, while a graft refers to tissue removed from its donor site without its native blood supply and relies on establishing new circulation through a process termed “take.” This process consists of two separate 48-hour phases: imbibition is the initial phase in which the graft is directly absorbing nutrients from the graft recipient bed; this is followed by inosculation, during which new blood supply is established.

### 1.2. Blood Supply to the Urethra and Penile Skin

**Figure 1:**
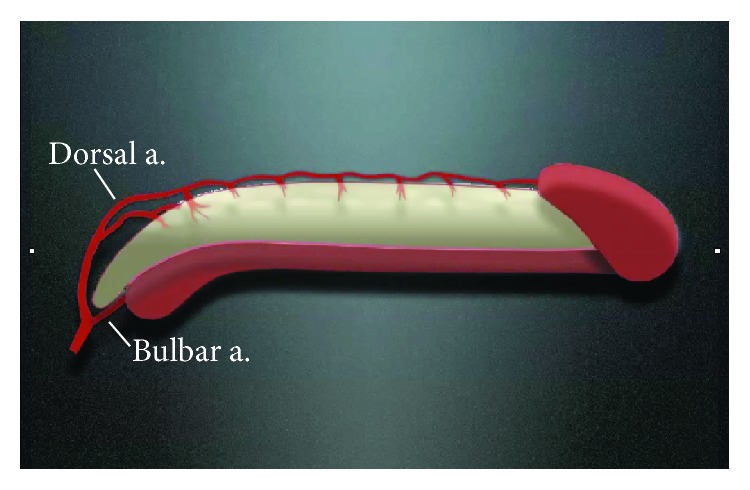
The dual blood supply to the urethra.

Detailed knowledge of the blood supply to the penile skin and corpus spongiosum is mandatory for successful tissue transfer. The healthy urethra within the corpus spongiosum has dual blood supply: it receives antegrade flow directly from the paired bulbar arteries and retrograde flow from the terminal branches of the dorsal arteries, which communicates with the corpus spongiosum in the glans penis (Figure 1). Although there are also small perforating vessels between the corpora cavernosa and corpus spongiosum, this is a minor contribution. This robust dual blood supply all within the corpus spongiosum allows aggressive mobilization of the spongiosum off of the corporal bodies without compromising the blood supply to the urethra. However, the distal blood supply to the corpus spongiosum is compromised in cases of hypospadias, especially more severe forms, or after prior repair and after urethroplasty. In these cases, wide mobilization may compromise the blood supply to the urethra and create ischemic stenosis.

**Figure 2:**
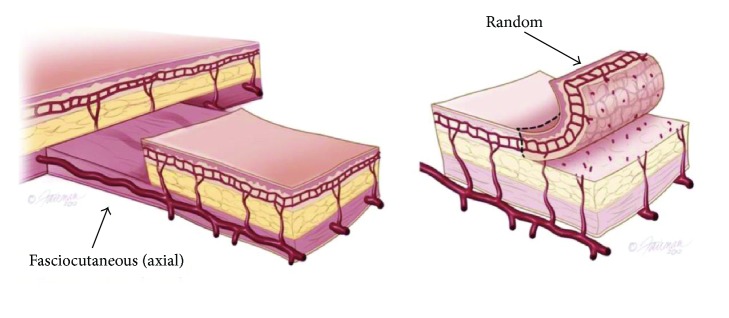
Axial and random flaps.

The penile skin receives its blood supply from branches of the superficial external pudendal artery. These branches travel just underneath the dartos fascia in an axial pattern, which provides reliable blood flow to skin flaps that are elevated on this fascial layer; hence these are referred to as fasciocutaneous flaps. There is also random blood supply achieved through the subdermal plexus, although this is much less dependable and not ideal for the survival of the flap (Figure 2).

### 1.3. Graft Material

The use of grafts in urethral reconstruction has been described since the late 19th century but was not popularized until Devine et al. began using full thickness penile skin grafts in 1961 [1]. This “patch graft” technique was historically the substitution procedure of choice, although it has now largely been supplanted by buccal mucosal grafts (BMG), split thickness skin grafts, and, in some cases, lingual grafts.

Buccal mucosal grafts have many advantages over penile skin and other materials which have led to their widespread use in recent years. These grafts are readily available and easily harvested and have more favorable vascular characteristics, including a rich submucosal plexus that facilitates good take. Additionally, buccal mucosa is nonhirsute and has an epithelial surface that is already well suited to a “wet” environment. As would be expected, long-term success rates with the use of BMG appear to be superior to penile skin grafts [2]. The use of lingual mucosa, while not as commonly used, is very similar in histology to buccal mucosa and has been described with very similar success rates [3].

### 1.4. Flap Techniques

Penile skin flaps, when used correctly, are a reliable and time-tested tool for urethral reconstruction. In the absence of prior flap surgery, penile skin (foreskin and distal penile skin in particular) is nonhirsute, has reliable axial vascular supply, and can be well mobilized and used to cover long urethral defects. Various flaps have been described, which can be elevated from ventral or dorsal skin and taken in either the longitudinal or transverse direction.

Orandi first described his ventral, longitudinal flap for penile urethral strictures using a lateral pedicle in 1968 with good results [4]. One disadvantage of this type of flap, however, is that if the stricture involves the proximal part of the pendulous or any part of the bulbar urethra, hair-bearing skin is involved in the reconstruction which can lead to recurrent infections and stone formation. For strictures isolated to the fossa, Jordan reported the use of a smaller, ventral penile skin flap that can be rotated onto the incised urethral opening [5].

For longer strictures, Quartey and McAninch have both described methods of obtaining transverse penile/preputial island flaps [6, 7]. These transverse flaps are versatile and hairless and can supply enough tissue to cover near panurethral defects. Furthermore, they involve a circumcision type incision with minimal disfigurement of the penis.

### 1.5. Grafting Techniques

Several techniques have been described, the two most common of which are the dorsal and ventral onlay grafts. The dorsal onlay approach was first described by Monseur in 1980 in which he incised the dorsal surface of the strictured urethra and sutured the edges directly to the corpora cavernosa to heal by secondary intention; this was later modified by Barbagli who used a penile skin or buccal graft to fill the defect [8, 9]. The ventral onlay graft was first described by Devine with the use of a full-thickness “patch graft” of penile skin and then later was modified to use with buccal mucosa [1, 10]. Several other grafting techniques have been described and will be detailed throughout this paper.

## 2. Selecting the Right Technique

Selecting the appropriate technique for each patient is highly individualized and dependent on multiple factors. The optimal repair will depend on the length and location of the stricture, the presence or absence of healthy, abundant penile skin, and whether or not the corpus spongiosum is intact. Incorporating all of these considerations can make the decision-making process quite complex; however, the proper selection of tissue transfer technique is paramount to success. Our aim is to provide a logical, easily comprehensible approach to the appropriate selection of grafts and flaps in urethral reconstruction.

## 3. Tissue Transfer to the Glans and Fossa Navicularis

**Figure 3:**
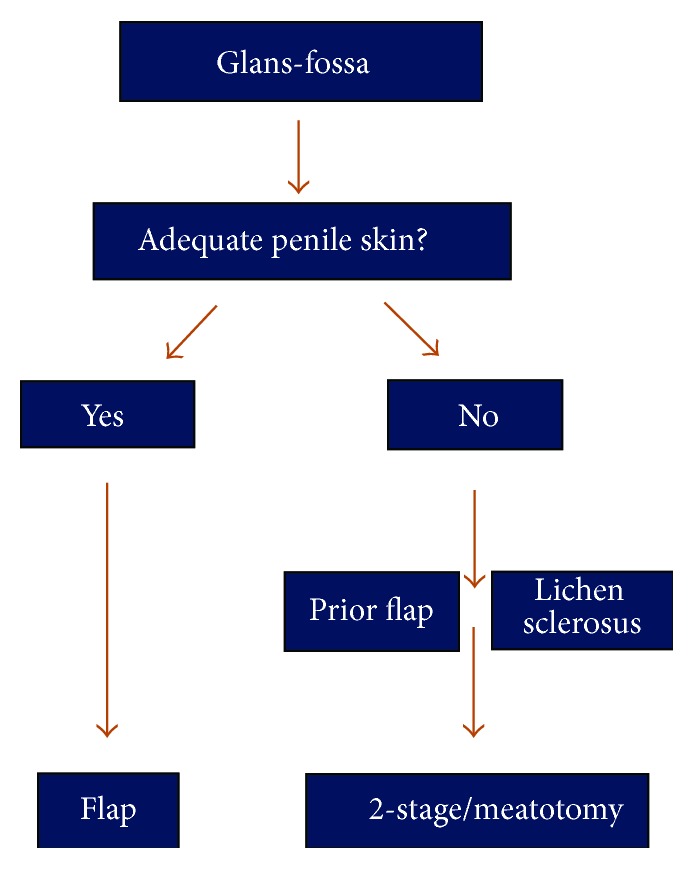
Treatment algorithm for strictures of the fossa navicularis.

Our approach to strictures of the glans and fossa navicularis is summarized in Figure 3. If a stricture is truly limited to the glans penis alone (meatal stenosis), a simple meatotomy is the procedure of choice. However, distal strictures often either extend into the fossa navicularis or are limited to the fossa. These strictures are often best treated with a one-stage flap repair as long as there is abundant and healthy penile skin. When this is not the case, as in cases of prior penile flap surgery or in cases of lichen sclerosus (LS) also known as balanitis xerotica obliterans (BXO), then a two-stage repair with buccal mucosa grafting is more appropriate given the prohibitively high recurrence rates when using skin in these situations [11, 12]. Alternatively, the patient may simply elect for an extended meatotomy if he believes that the resulting ventral displacement of the meatus is cosmetically acceptable and prefers a simple procedure.

**Figure 4:**
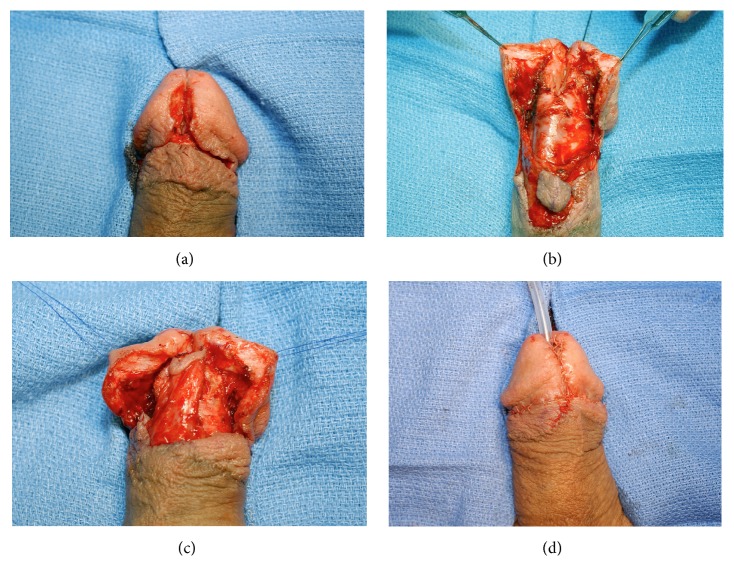
(a) An extended meatotomy is made through the stricture. (b) Glans wings are mobilized and a ventral flap is isolated. (c) The ventral flap has been rotated onto the defect. (d) Immediate appearance after closure.

Although multiple flap techniques have been described, we prefer the ventral transverse island flap as initially described by Jordan for this location [5]. This technique involves incising the urethra ventrally through the stricture, elevating a transverse skin island on a broad pedicle of dartos fascia, and inverting the flap onto the defect prior to closure (Figure 4). This technique has been demonstrated to be highly successful, with Jordan reporting success in all 23 patients who did not have LS with an average follow-up of 10 years. Of note, the success was only 50% (6/12 patients) in those who had LS, reaffirming the recommendation against using penile skin in these cases [12].

Grafts are used by some authors for one-stage repairs of strictures involving the glans and fossa navicularis with dorsal graft placement [13]. Others place buccal mucosa grafts as ventral onlays using the glans wings as the graft bed [14, 15]. However, we do not believe that these techniques in our hands obtain the same caliber of patency (24–30 French) that can be achieved with ventral flaps or staged repairs. Most importantly, however, it should be emphasized that the priority of the repair is relief of obstruction; thus the caliber of the repair, regardless of technique, should not be compromised by aggressively attempting to bring the meatus all the way to the tip of the glans.

## 4. Tissue Transfer to the Penile Urethra and Bulbar Urethra

**Figure 5:**
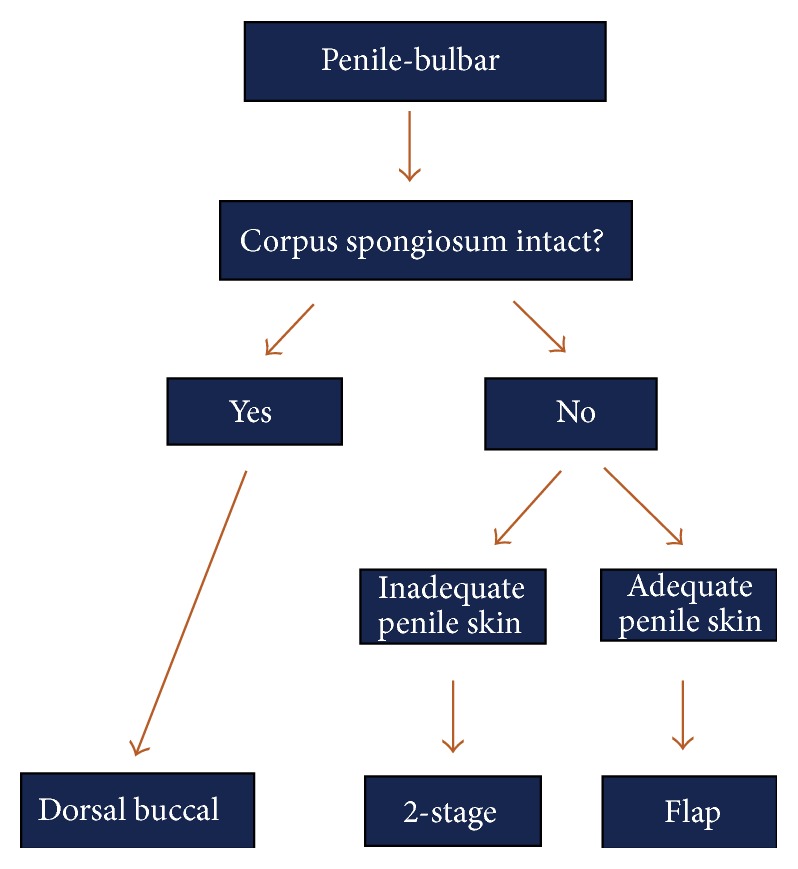
Treatment algorithm for strictures of the penile urethra and bulbar urethra.

The repair of penile and bulbar urethral strictures that are not amenable to EPA can be performed with grafts, flaps, or staged procedures, depending on whether the corpus spongiosum is intact and whether there is sufficient penile skin, as summarized in the algorithm in Figure 5. There is some debate about at what length an EPA should be avoided in favor of a tissue transfer technique, as lengthy excisions can lead to a tethered penis and tension on the anastomosis. In general, strictures in the penile urethra and distal bulbar urethra are only highly amenable to EPA when they are short (i.e., less than 2 cm), whereas primary repairs without undue tension can be achieved for longer proximal bulbar strictures. In this location, maneuvers including separation of the corporal bodies and detachment of the bulb from the perineal body are options, and EPAs have been described for strictures up to 5 cm long in this location [16].

### 4.1. When the Corpus Spongiosum Is Intact

There is general consensus that strictures of the penile urethra not amenable to excisional repair are best repaired with a dorsal onlay graft, as the spongiosum even when intact is tenuous in this area and does not supply a reliable vascular bed to a ventrally placed graft [17]. Prior to the popularization of BMG, skin flaps were preferred as the most reliable approach when available. Dorsally placed buccal grafts, however, have also been demonstrated in multiple studies to provide very reliable results and are more durable and better suited to the “wet” environment than penile skin [17, 18]. Moreover, with the use of dorsal buccal grafting, the dorsal aspect of the urethra is supported by the corporal bodies, and the ventral and lateral native urethra is supported by intact corpus spongiosum, which will likely prevent both fistula formation and diverticular change.

Strictures of the bulbar urethra have generated considerably more debate as to the optimal location and technique of graft placement. There is an anatomical difference between the penile urethra and bulbar urethra with regard to the ventral spongiosum. As the urethra moves proximally, it becomes more dorsally located so that the spongiosum becomes thicker and more robust ventrally, thus providing a potentially suitable vascular bed for graft take.

Some authors prefer the ventral approach as it limits urethral mobilization with preservation of cavernosal-spongiosal perforating arteries [19]. In addition, the ventral approach is often considered to be less technically challenging with shorter operative times. Even Barbagli, who developed the dorsal onlay graft, has noted preference for the ventral approach in certain situations: if the dorsal aspect of the urethra is scarred down to the corpora from prior surgery or if the stricture extends proximally beyond the triangular ligament [20].

However, several advantages exist to the dorsal approach, including the fact that the graft can be spread fixated to the corpora cavernosa, which supplies a consistently reliable graft bed that is not affected by spongiofibrosis. This spread-fixation, which cannot be accomplished with the ventral approach, also maximizes the surface area of the graft that is in direct contact with its vascular bed. This optimizes the conditions for graft take and allows for a widely patent lumen, ideally up to 30 French. Additionally, the use of a dorsal approach may reduce the incidence of postvoid dribbling, which has been shown in at least one retrospective study to be more prominent with the ventral approach [21].

Fueling the controversy is the fact that there have not been any randomized controlled trials to compare these two techniques, and the evidence that does exist is limited by its retrospective nature and conflicting results. One early comparison of 71 patients concluded that the dorsal onlay method was superior (5% versus 14% failure rates), but no statistical analysis was performed [22]. In contrast, Barbagli et al. retrospectively compared 17 ventral, 27 dorsal, and 6 lateral bulbar urethroplasties and found a success rate (defined by lack of subsequent treatment at mean follow-up of 42 moths) of 83%, 85%, and 83%, respectively [21]. More recently, a group of authors published a multicenter series with extended follow-up (median of 118 months) and found very similar success rates between the two techniques (80.2% of 81 patients with dorsal onlay compared to 81.5% of 130 patients with ventral onlay (*n* = 130)) [2]. However, we advise caution before concluding that these results are equivalent because each patient was carefully selected for the procedure they received and therefore the inherent selection bias prohibits this study from providing a decisive comparison. Another large retrospective study compared 62 ventral onlay cases with 41 dorsal onlay cases and reported equivalent outcomes with failure rates of 19% and 17%, respectively, at a mean follow-up of 36 months. However, the authors admit a selection bias as dorsal onlay procedures are reserved for more complicated strictures at their institution [19]. A recent systematic review of published series showed very little difference between the two techniques. The published ventral onlay series success rates ranged from 83% to 100% with an average of 88.84%, while the reviewed dorsal onlay series successes ranged from 73% to 100% with an average of 88.37% [17].

An additional technique was described in 2001 by Asopa et al., in which they were able to access the dorsal aspect of the urethra via a ventral urethrotomy and then excise an elliptical portion of the dorsal urethra and apply a dorsal “inlay” graft in its place before retubularizing the urethra [23]. The benefit of this approach is to combine the advantages of having a dorsally placed graft laying on the corporal graft bed while avoiding division of the perforating arteries that occurs during urethral mobilization to maintain maximal blood supply. This technique has been evaluated by several authors with results that compare to the historical results for the ventral and dorsal onlay approaches [17].

While the debate between these techniques is likely to continue until higher level evidence emerges, we prefer the dorsal approach at our institution for the variety of reasons mentioned above.

### 4.2. When the Corpus Spongiosum Is Not Intact

In cases where the spongiosum is not intact, such as in hypospadias, a one-stage graft is not recommended as the blood supply to the urethra will be severely compromised once it is fully mobilized. In these cases, whether it involves the penile or bulbar urethra, a skin flap is more appropriate as long as there is adequate and healthy penile skin. Transverse fasciocutaneous penile/preputial skin flaps can provide excellent coverage and have achieved good to excellent results in the published literature. In his initial description, McAninch obtained flaps up to 15 cm with no stricture recurrence in 10 patients with strictures which are 8–21 cm long and a mean follow-up of 14 months [24]. A subsequent publication of his long-term data revealed a success rate of 87% in 54 patients [25]. A review by Wessells and McAninch evaluated nine studies, all with at least one year of follow-up, and found that success rates ranged from 77% to 95%, with an average of 85.5% [18].

**Figure 6:**
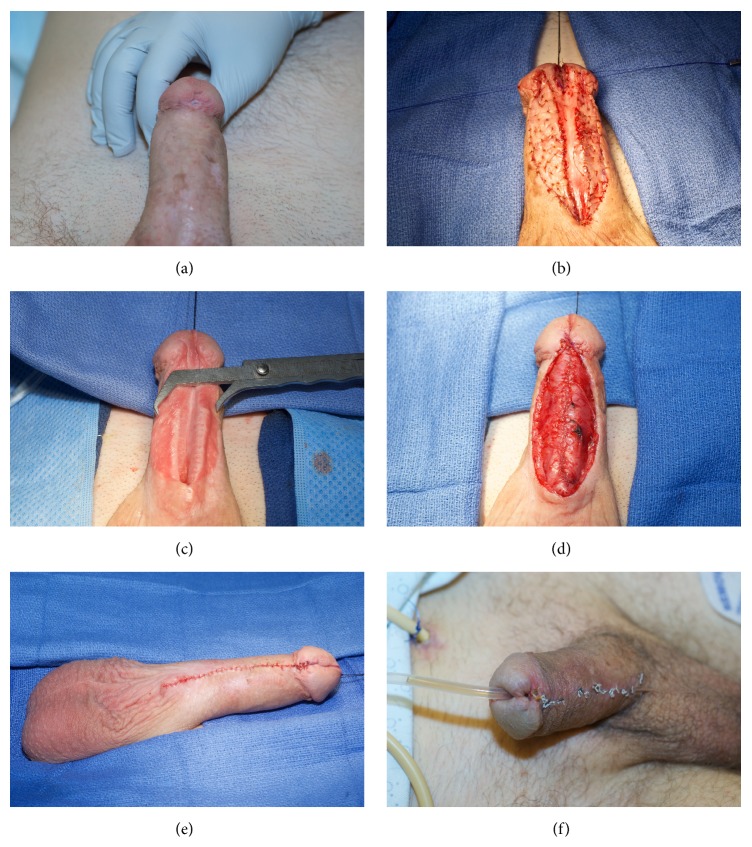
A 2-stage repair performed for a patient with a fossa and penile urethral stricture. (a) Demonstration of inadequate penile skin. (b) BMG quilted on either side of the opened urethral plate. (c) The urethral plate is now very adequate after healing of 1st  stage. (d) Tubularization of new urethral plate. (e and f) Postoperative appearance immediately after closure and 3 weeks postoperatively.

When there is not enough healthy skin, such as in cases of LS or prior flap surgery, a two-staged approach is more appropriate (Figure 6).

## 5. Special Situations

In certain complex cases with a segment of obliterated or near-obliterated urethra, there is not an adequate urethral plate to perform a ventral or dorsal onlay graft. If these are short strictures, they are best treated by excision with primary anastomosis (EPA). However, in such cases when the stricture is too long for an EPA, an augmented anastomosis can be considered. This technique, initially described by Turner-Warwick, involves excising the stricture, placing a graft dorsally, and then reanastomosing the native urethral edges ventrally [26]. Yet even this technique can be limited by length, with the longest stricture treated with this technique in one prominent series being of only 2 cm [27].

In patients with longer obliterative segments, an EPA or augmented anastomotic repair may not be feasible. Additionally, in patients who have already failed urethroplasty or have a history of hypospadias, strictures that would otherwise be technically amenable to one of these repairs may be at risk of urethral ischemia with urethral transection. For this small subset of patients, a more involved and creative approach may be necessary. Tabularized grafts and flaps have been attempted but have significantly high failure rates, reportedly up to 58%; therefore other techniques are needed to repair these challenging cases [14, 28].

### 5.1. Graft/Flap Combination

**Figure 7:**
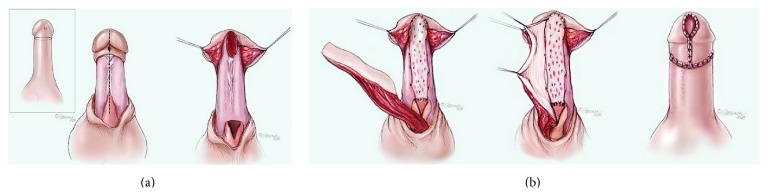
Graft/flap combination. (a) The obliterated urethra is incised proximally until a healthy, widely patent lumen is encountered. (b) The buccal graft is spread fixated to the corpora cavernosa, after which a penile skin flap is rotated ventrally onto the graft to create a new lumen. In cases where there is a deficiency of urethra within the fossa and a lack of a groove within the glans penis, a defect is created and the BMG is extended into the glans.

A more successful method to treat these strictures is with the combination of a dorsal buccal graft to augment or replace the inadequate urethral plate, followed by a penile skin flap onlay reconstruction (Figure 7). This was initially described by Morey in 2001 for single stage circumferential tissue transfer in 2 patients with penile urethral strictures [29]. A larger series of 12 patients was subsequently published with a success rate of 92% defined as wide patency documented by cystoscopy 4 months after surgery with subsequent follow-up that averaged 39 months [30].

The graft and flap combination can also be used for panurethral strictures that are too long for repair with BMG even when bilateral grafts are harvested. This technique typically involves using as much BMG as possible in the proximal aspects of the stricture and then using a penile skin flap to repair the remainder of the stricture distally [31, 32].

### 5.2. Combined Dorsal/Ventral Buccal Grafting

**Figure 8:**
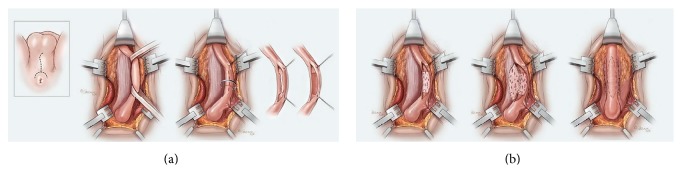
Dorsal and ventral buccal graft combination. (a) The urethra is mobilized and incised dorsally and healthy ventral spongiosum is exposed. (b) A buccal graft is spread fixated to the spongiosum where the obliterated segment was located, and an additional buccal graft is applied to the corpora cavernosa before the edges are anastomosed for retubularization.

If penile skin is not available for a flap, then a combination of a dorsal and ventral BMG may be used. Such a combination approach was initially described by Palminteri et al. who used the Asopa technique to place a dorsal inlay graft and then place a ventral onlay graft in the ventral urethrotomy to obtain additional area within the new lumen [33]. However, this description was not targeted to obliterative strictures as the technique relies on a native urethra wide enough to be sutured to both grafts. Gelman and Siegel recently reported results from our institution on a series of 18 patients who had segments of total or near-total obliteration of their urethras and underwent combined ventral and dorsal buccal grafting for a 1-stage repair. The technique involves a dorsal incision without transection of the mobilized urethra, thereby preserving the continuity of the blood supply within the spongy tissue. Buccal mucosa is quilted dorsally to the corporal bodies in the standard dorsal onlay fashion. Additional buccal mucosa can then be quilted to the dorsally incised, nontransected corpus spongiosum in continuity with the distally and proximally spatulated urethra. The repair is then completed by approximating dorsal and ventral buccal mucosal graft segments (Figure 8). We feel the strengths of this technique include being able to leave the robust ventral spongy tissue intact and being able to place quilting sutures to secure the graft firmly to its bed. In this series with a mean follow-up of 50 months, the success rate was 94% (100% after the single failure underwent an internal urethrotomy) [34]. Although this needs to be validated by other studies, it remains a promising technique for some of the most challenging cases.

## 6. Summary

The use of grafts and flaps in the treatment of urethral stricture disease remains an indispensable tool in the armamentarium of the reconstructive urologist. While success rates are very difficult to compare between various techniques at this time, all of the current techniques mentioned appear to be highly successful for appropriately selected patients. The decision on which technique to use is dependent on a variety of factors. For strictures involving the fossa navicularis, the use of a penile skin flap provides excellent coverage while leaving a widely patent lumen. A meatotomy or two-stage repair should be considered if the penile skin is unhealthy or deficient. For strictures of the penile or bulbar urethra, we prefer the dorsal buccal approach as long as the corpus spongiosum is intact and reserve the use of a flap for when the spongiosum is not intact or a two-stage repair if the penile skin will not allow a flap to be used. Randomized controlled trials will likely be necessary to definitively recommend one technique over another, but until that time, it is imperative for the surgeon to be comfortable with all of the described techniques to individualize the treatment approach for each patient.
